# 洛铂与顺铂胸腔灌注化疗治疗恶性胸腔积液的疗效及不良反应的*Meta*分析

**DOI:** 10.3779/j.issn.1009-3419.2019.02.03

**Published:** 2019-02-20

**Authors:** 诗惠 闵, 强强 郑, 白露 张, 丹丽 严, 汝兰 王, 子涵 瞿, 潞 李, 洁薇 刘, 清华 周

**Affiliations:** 1 610041 成都，四川大学华西医院肺癌中心 Lung Cancer Center, West China Hospital, Sichuan University, Chengdu 610041, China; 2 300052 天津，天津医科大学总医院天津市肺癌研究所 Lung Cancer Institute, Tianjin Medical University General Hospital, Tianjin 300052, China

**Keywords:** 洛铂, 顺铂, 恶性胸腔积液, *Meta*分析, Lobaplatin, Cisplantin, Malignant pleural effusion, *Meta*-analysis

## Abstract

**背景与目的:**

系统评价洛铂（Lobaplatin, LBP）和顺铂（Cisplatin, DDP）胸腔灌注化疗治疗恶性胸腔积液（malignant pleural effusion, MPE）的疗效和不良反应。

**方法:**

计算机检索Medline（PubMed）Embase、Web of Science、Cochrane、万方数据库、中国期刊全文数据库（CNKI）和维普数据库（VIP），收集有关LBP与DDP胸腔灌注化疗治疗MPE的随机对照试验（randomized control trial, RCT）；主要结局指标包括客观缓解率（objective response rate, ORR）、完全缓解率（complete response, CR）、部分缓解率（partial response, PR）、肾毒性、胸痛、胃肠道反应、骨髓抑制、发热反应及肝毒性；采用相对危险度（relative risk, RR）为效应量，各效应量以95%置信区间（95%CI）表示，Stata 14.0统计软件进行*meta*分析。

**结果:**

共纳入12项RCT，共720例MPE患者，*meta*分析结果显示，与DDP胸腔灌注化疗方案比较，LBP胸腔灌注化疗方案明显提高MPE患者的ORR（RR=1.27, 95%CI: 1.15-1.40, *P*<0.001）、CR（RR=1.39, 95%CI: 1.09-1.78, *P*=0.007）、PR（RR=1.21, 95%CI: 1.02-1.42, *P*=0.026）；与DDP胸腔灌注化疗比较，LBP胸腔灌注化疗组的肾毒性（RR=0.31, 95%CI: 0.13-0.71, *P*=0.005）、胃肠道反应（RR=0.44, 95%CI: 0.31-0.62, *P* < 0.001）发生率显著低于DDP。

**结论:**

与DDP胸腔灌注化疗治疗MPE比较，LBP胸腔灌注化疗治疗MPE的ORR、CR和PR均显著优于DDP，且肾毒性及胃肠道反应均显著低于DDP。

## Abstract (en)

**Background and objective:**

The aim of this study is to systematically evaluate the efficacy and adverse effects of Lobaplatin and Cisplatin in the treatment of malignant pleural effusion.

**Methods:**

The databases of Medline (PubMed), Embase, Web of Science, Cochrane, Wanfang, CNKI and VIP were retrieved so as to search the studies about the randomized controlled clinical trials (RCT) that compared the Lobaplatin and Cisplatin for malignant pleural effusion. The main outcome indicators include objective response rate, complete response, partial response, nephrotoxicity, chest pain, gastrointestinal reaction, myelosuppression, fever response and hepatotoxicity. Relative risk was used as the effect size, which was expressed as 95% confidence interval. The *meta*-analysis was performed using Stata 14.0 statistical software.

**Results:**

A total of 12 RCTs and 720 MPE patients were included. The results showed that the ORR (RR=1.27, 95%CI: 1.15-1.40, *P* < 0.001), CR (RR=1.39, 95%CI: 1.09-1.78, *P*=0.007), PR (RR=1.21, 95%CI: 1.02-1.42, *P*=0.026) in LBP thoracic perfusion chemotherapy were significantly higher than those in DDP thoracic perfusion chemotherapy. The incidence of nephrotoxicity (RR=0.31, 95%CI: 0.13-0.71, *P*=0.005) and gastrointestinal reactions (RR=0.44, 95%CI: 0.31-0.62, *P*< 0.001) in the LBP group were significantly lower than those in DDP group.

**Conclusion:**

Compared with DDP pleural perfusion chemotherapy, the ORR, CR and PR of LBP pleural perfusion chemotherapy for MPE are significantly better than DDP, and its nephrotoxicity and gastrointestinal reactions are remarkably lower than DDP.

恶性胸腔积液（malignant pleural effusion, MPE）是指在胸腔积液或胸膜活检中发现恶性细胞，多由原发性胸膜恶性肿瘤或伴有胸膜转移的肿瘤引起，是临床上晚期恶性肿瘤患者常见的问题，仅美国每年的MPE发病率据估计至少15万例^[1]^。据估计，15%的各类癌症患者会因原发癌症的胸膜转移而发生恶性胸腔积液^[2]^。第8版肺癌TNM分期中将MPE分期为M1a，预示着MPE患者预后差^[3]^，中位生存期在6个月-18个月^[4]^，且MPE患者缺乏标准的治疗方法^[5]^。目前，MPE的治疗方法包括胸腔积液引流、胸膜腔内化疗和全身化疗，但这些方法通常是姑息性的，目的是减轻肺受压症状，减轻胸部不适，提高患者的生活质量，但其治疗效果仍不尽人意。

胸膜腔内灌注化疗是一种临床上比较常用的治疗恶性胸腔积液的方法，通过将化疗药物如顺铂（Cisplatin, DDP）灌注入胸腔起到杀灭肿瘤细胞的作用^[6]^。Tan等^[6]^报道MPE患者在手术干预后接受胸腔灌注化疗后其中位生存15.4个月。目前常用的灌注化疗药物多为卡铂（Carboplatin, CBDCA）和DDP^[7]^、洛铂（Lobaplatin, LBP）等化疗药物。LBP是新一代铂类抗肿瘤药物，具有水溶性好、抗瘤活性强、与其他铂类药物无交叉耐药性及毒副作用低等特点^[8]^。但尚缺乏对两者比较的系统性评价，本研究旨在系统性评价LBP与DDP胸腔内灌注化疗治疗MPE的疗效以及不良反应。

## 1 资料与方法

### 1.1 纳入标准

#### 1.1.1 研究类型

公开发表的随机对照试验（randomized control trial, RCT），无论是否采用盲法和分配隐藏，语种限定为中文文献，发表日期截止于2018年12月31日。

#### 1.1.2 研究对象

经病理学或细胞学，以及影像学诊断证实的恶性胸腔积液的患者。

#### 1.1.3 干预措施

接受LBP胸腔灌注化疗或DDP胸腔灌注化疗。

#### 1.1.4 结局指标

客观缓解率（objective response rate, ORR）、完全缓解率（complete response, CR）、部分缓解率（partial response, PR）以及肾毒性、胸痛、胃肠道反应、骨髓抑制、发热、肝毒性等不良反应发生率。

### 1.2 排除标准

① 非RCT研究；②综述性文献；③重复发表的文献；④同时接受放疗或者其他治疗方案的研究；⑤当多篇涉及同一研究时，以最近发表的文献为准。

### 1.3 文献检索策略

计算机检索Medline（PubMed）、Embase、Web of Science、Cochrane、万方数据库，中国期刊全文数据库（CNKI）和维普数据库（VIP），收集关于LBP与DDP胸腔灌注化疗治疗MPE的RCT。检索词主要包括：恶性胸腔积液、胸腔积液、胸水、化疗、顺铂、洛铂、胸腔灌注化疗和灌注化疗；对纳入文献的参考文献进一步扩大检索。

### 1.4 文献筛选和资料提取

由两位研究者独立阅读检索出的文章题目和摘要，在排除明显不符合纳入标准的试验后，对可能符合纳入标准的试验进行全文阅读，以明确是否符合纳入标准，两位研究人员交叉核对纳入试验的结果，对有分歧而难以确定的试验通过讨论决定其是否纳入，提取信息主要包括：①文章题目、作者、发表日期及来源等；②患者的性别、年龄、病理类型、TNM分期及具体干预措施等；③结局指标，包括ORR、CR、PR及肾毒性、胸痛、胃肠道反应、骨髓抑制、发热、肝毒性等不良反应发生率。如果临床试验报告不详或资料缺乏，通过电话或信件等方式与作者联系予以补充。文献纳入、数据提取均2名研究人员独立完成。

### 1.5 质量评价

纳入文献采用Jadad量表^[9]^进行质量评分，总分1分-2分为低质量文献，3分-5分为高质量文献。质量评价均2名研究人员独立完成。

### 1.6 统计学分析

采用STATA 14.0软件进行*meta*分析，计数资料采用相对危险度（relative risk, RR）及其95%CI表示。各纳入研究结果间的异质性采用卡方检验。当各研究间有统计学同质性（*P* > 0.1且*I*^2^ < 50%）时，采用固定效应模型对各研究进行*meta*分析；如各研究间存在统计学异质性（*P* < 0.1或*I*^2^ > 50%）时，分析其质性来源，必要时对可能导致异质性的因素进行亚组分析。用*Begg’s*检验和*Egger’s*检验检测潜在的发表偏倚，*Begg’s*检验漏斗图的不对称性以及*Egger’s*检验*P* < 0.05或*t*值得95%CI不包括0均表示存在发表偏倚。

## 2 结果

### 2.1 文献检索结果及质量评价

从中英文数据库初检出相关中文文献629篇，通过阅读文献题目和摘要，排除细胞实验、动物实验、综述、病例报告、单药的临床观察、腹腔灌注化疗、结合中药治疗等不符合条件的文献，得到14篇文献。进一步阅读全文，排除特殊人群、非随机对照试验及重复研究，最终纳入12个研究^[10-21]^。文献筛选流程及结果见图 1。12个RCT共纳入720例患者（LBP组393例，DDP组327例），纳入分析的文献两组基线资料均具有可比性，其基本特征见表 1，纳入研究的质量评价结果（表 1）。

**1:**
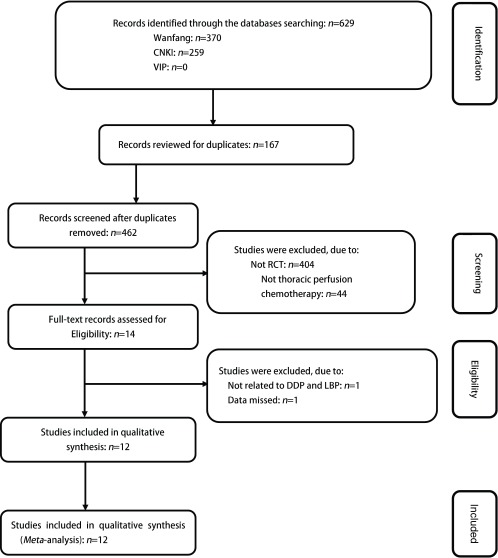
文献检索及筛选流程图 Document retrieval and screening flow chart

**1:** 纳入本*meta*分析的12项研究的基本资料及质量评价 Basic data and quality evaluation of the 12 studies included in the *meta*-analysis

First author (year)	Time of enrollment	No. of LBP	No. of DDP	Experimental group drug	Control group drug	Jadad scores
Qianqian Feng (2015)	2012.01-2013.12	40	40	LBP	DDP	2
Qiurong Huang (2017)	2014.04-2016.04	38	38	LBP	DDP	3
Xianguang Liu (2013)	2009.05-2012.08	30	30	LBP	DDP	3
Xuejun Liu (2016)	2013.01-2015.12	30	30	LBP	DDP	2
Hailong Ma (2017)	2014.01-2016.12	30	30	LBP	DDP	2
Zhihong Sheng (2014)	-	30	30	LBP	DDP	2
Xiaoqiang Sun (2012)	2010.01-2011.06	27	25	LBP	DDP	2
Yue Wang (2016)	2013.01-2015.07	30	29	LBP	DDP	2
Ming Zhang (2018)	2016.10-2017.05	25	27	LBP	DDP	2
Lu Zhao (2017)	2013.11-2015.11	14	16	LBP	DDP	2
Haiyan Xu (2013)	2007.11-2011.12	75	71	LBP	DDP	2
Chao Li (2015)	2012.01-2014.04	24	23	LBP	DDP	2

### 2.2 *Meta*分析结果

#### 2.2.1 ORR

纳入的12个研究均报告了ORR，各研究间具有同质性（*P*=0.735, *I-squared*=0.0%），采用固定效应模型进行*meta*分析，结果显示：LBP组ORR显著高于DDP组，其差异具有统计学意义（RR=1.27, 95%CI: 1.15-1.40, *P* < 0.001）（表 2、图 2A）。

**2:** 洛铂与顺铂胸腔灌注化疗治疗恶性胸腔积液患者的疗效和不良反应的*meta*分析结果 *Meta*-analysis of efficacy and adverse reactions of LBP and DDP intrapleural infusion chemotherapy in patients with MPE

Outcome indicators	*n*	Effects model	RR (95%CI)	*P*	Publication bias	Statistical differences
					Egger (P) (95%CI of *t* value)	
ORR	12	Fixed	1.27 (1.15-1.40)	< 0.001	0.274 (-1.05-3.33)	Significant
CR	12	Fixed	1.40 (1.09-1.78)	0.007	0.556 (-0.79-1.38)	Significant
PR	12	Fixed	1.21 (1.02-1.42)	0.026	0.958 (-2.02-2.12)	Significant
Nephrotoxicity	3	Fixed	0.31 (0.13-0.71)	0.005	0.697 (-25.94-23.91)	Significant
Chest pain	6	Fixed	0.89 (0.67-1.18)	0.413	0.373 (-2.32-1.09)	Non-significant
Gastrointestinal reaction	8	Random	0.44 (0.31-0.62)	< 0.001	0.004 (-5.18--1.60)	Significant
Myelosuppression	5	Random	0.85 (0.56-1.29)	0.431	0.262 (-4.28-1.69)	Non-significant
Fever	5	Fixed	0.92 (0.61-1.40)	0.702	0.711 (-1.90-1.47)	Non-significant
Hepatotoxicity	2	Fixed	0.82 (0.36-1.86)	0.631	-	Non-significant
ORR: objective mitigation rate; CR: complete response; PR: partial response; RR: relative risk; CI: confidence interval; LBP: Lobaplatin; DDP: Cisplatin; MPE: malignant pleural effusion.						

**2:**
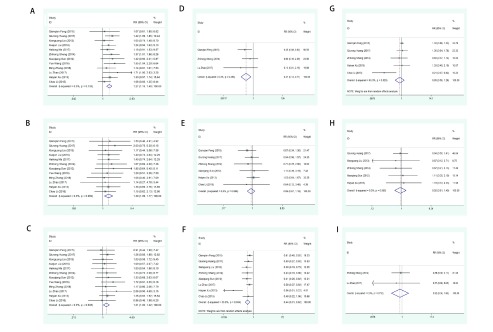
DDP与LBP胸腔灌注化疗治疗MPE疗效及不良反应的*meta*分析森林图。A：ORR的*meta*分析结果显示LBP组ORR显著高于DDP组；B：CR的*meta*分析结果显示，LBP组CR显著优于DDP组；C：PR的*meta*分析结果显示LBP组PR显著优于DDP组；D：肾毒性发生率*meta*分析结果显示，DDP组肾脏毒性发生率显著高于LBP组；E：胸痛发生率*meta*分析结果显示DDP组与LBP组胸痛发生率无统计学差异；F：胃肠道反应发生率*meta*分析结果显示DDP组胃肠道反应发生率显著高于LBP组；G：骨髓抑制发生率*meta*分析结果显示DDP组与LBP组间骨髓抑制发生率无统计学差异；H：发热反应*meta*分析结果显示DDP组与LBP组间发热反应发生率无统计学差异；I：肝毒性*meta*分析结果显示DDP组与LBP组间肝毒性发生率无统计学差异 Forest maps of *meta*-analysis of comparing the efficacy and adverse reactions of DDP and LBP thoracic perfusion chemotherapy for MPE. A: The *meta*-analysis of ORR showed that ORR in LBP group was significantly higher than that in DDP group; B: The *meta*-analysis of CR showed that CR in LBP group was significantly better than that in DDP group; C: The *meta*-analysis of PR showed that PR in LBP group was significantly better than that in DDP group; D: The *meta*-analysis of nephrotoxicity showed that the incidence of nephrotoxicity in DDP group was significantly higher than that in LBP group; E: The *meta*-analysis of chest pain showed that the incidence of chest pain in DDP group and LBP group werenot significantly different; F: The *meta*-analysis of incidence of gastrointestinal reaction showed that in DDP group was significantly higher than that in LBP group; G: The *meta*-analysis ofthe incidence of myelosuppressionshowed that in DDP group was not significantly different from that in LBP group. H: Meta-analysis of fever reaction showed that there was no significant difference in the incidence of fever reaction between DDP group and LBP group. I: The results of *meta*-analysis showed that there was no significant difference in the incidence of hepatotoxicity between DDP group and LBP group

#### 2.2.2 CR

纳入的12个研究均报告了CR，各研究间具有同质性（*P*=0.999, *I-squared*=0.0%），采用固定效应模型进行*meta*分析，结果显示，LBP组CR显著优于DDP组，其差异具有统计学意义（RR=1.40, 95%CI: 1.09-1.78, *P*=0.007）（表 2、图 2B）。

#### 2.2.3 PR

纳入的12个研究均报告了PR，各研究间具有同质性（*P*=0.938, *I-squared*=0.0%），采用固定效应模型进行*meta*分析，结果显示，LBP组PR显著优于DDP组，其差异具有统计学意义（RR=1.21, 95%CI: 1.02-1.42, *P*=0.026）（表 2、图 2C）。

#### 2.2.4 肾毒性

纳入的12个研究中有3个研究报告了肾毒性发生率，对3篇文献进行统计分析，各研究间具有同质性（*P*=0.483, *I-squared*=0.0%），采用固定效应模型进行*meta*分析。比较LBP与DDP方案胸腔灌注化疗肾脏毒性发生率差异，结果显示：DDP组肾脏毒性发生率显著高于LBP组，差异具有统计学意义（RR=0.31, 95%CI: 0.13-0.71, *P*=0.005）（表 2、图 2D）。

#### 2.2.5 胸痛

纳入的12个研究中有6个研究报告了胸痛发生率，对6篇文献进行统计分析，各研究间具有同质性（*P*=0.898, *I-squared*=0.0%），采用固定效应模型进行*meta*分析，比较LBP与DDP方案胸腔灌注化疗胸痛发生率差异，结果显示：DDP组与LBP组间：胸痛发生率无统计学差异（RR=0.89, 95%CI: 0.67-1.18, *P*=0.413）。

#### 2.2.6 胃肠道反应

纳入的12个研究中有8个研究报告了胃肠道反应发生率，对8篇文献进行统计分析，各研究间具有异质性（*P*=0.034, *I-squared*=53.9%），采用随机效应模型进行*meta*分析，比较LBP与DDP方案胸腔灌注化疗胃肠道反应发生率差异，结果显示：DDP组胃肠道反应发生率显著高于LBP组，差异具有统计学意义（RR=0.44, 95%CI: 0.31-0.62，*P* < 0.001）。

#### 2.2.7 骨髓抑制

纳入的12个研究中有5个研究报告了骨髓抑制发生率，对5篇文献进行统计分析，各研究间具有异质性（*P*=0.029, *I-squared*=63.0%），采用随机效应模型进行meta分析，比较LBP与DDP方案胸腔灌注化疗骨髓抑制发生率差异，结果显示：DDP组与LBP组间骨髓抑制发生率无统计学差异（RR=0.85, 95%CI: 0.56-1.29, *P*=0.431）。

#### 2.2.8 发热反应

纳入的12个研究中有5个研究报告了发热反应的发生了，对5篇文献进行统计分析，各研究间具有同质性（*P*=0.950, *I-squared*=0.0%），采用固定效应模型进行*meta*分析，比较LBP与DDP方案胸腔灌注化疗发热反应发生率差异，结果显示：DDP组与LBP组间发热反应发生率无统计学差异（RR=0.92, 95%CI: 0.61-1.40, *P*=0.702）。

#### 2.2.9 肝毒性

纳入的12个研究中有2个研究报告了肝毒性，对2篇文献进行统计分析，各研究间具有同质性（*P*=0.732, *I-squared*=0.0%），采用固定效应模型进行*meta*分析。比较LBP与DDP方案胸腔灌注化疗肝毒性发生率差异，结果显示：DDP组与LBP组间肝毒性发生率无统计学差异（RR=0.82, 95%CI: 0.36-1.86, *P*=0.631）。

### 2.3 发表偏倚

漏斗图用于发现可能的发表偏倚，除了胃肠道反应的漏斗图不对称外，其余各漏斗图均对称（图 3）；*Egger’s*检验结果表明（表 2），在ORR（*t*=1.16, 95%CI: -1.05-3.33, *P*=0.274）、CR（*t*=0.61, 95%CI: -0.79-1.38, *P*=0.556）、PR（*t*=0.05, 95%CI: -2.02-2.12, *P*=0.958）、肾毒性（*t*=-0.52, 95%CI: -25.94-23.91, *P*=0.697）、胸痛（*t*=-1.00, 95%CI: -2.32-1.09, *P*=0.373）、骨髓抑制（*t*=-1.38, 95%CI: -4.28-1.69, *P*=0.262）以及发热（*t*=-0.41, 95%CI: -1.90-1.47, *P*=0.711）等结果的报道中不存在发表偏倚，而胃肠道反应（*t*=-4.63, 95%CI: -5.18-1.60, *P*=0.004）结果的报道存在发表偏倚。

**3:**
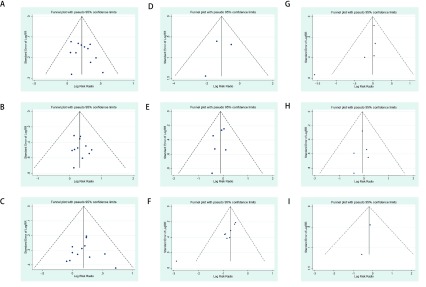
DDP与LBP胸腔灌注化疗治疗MPE疗效及不良反应的漏斗图。A：ORR的*meta*分析漏斗图左右对称；B：CR的*meta*分析结果漏斗图左右对称；C：PR的*meta*分析漏斗图左右对称；D：肾毒性发生率*meta*分析漏斗图左右对称；E：胸痛发生率*meta*分析漏斗图左右对称；F：胃肠道反应发生率*meta*分析漏斗图左右不对称；G：骨髓抑制发生率*meta*分析漏斗图左右对称；H：发热反应发生率*meta*分析漏斗图左右对称；I：肝毒性发生率*meta*分析漏斗图左右对称 Funnel plots of comparing the efficacy and adverse effects of DDP and LBP in thoracic perfusion chemotherapy for MPE. A:The funnel plot of *meta*-analysis of ORR is symmetry; B: The funnel plot of *meta*-analysis of CR is symmetry; C: The funnel plot of *meta*-analysis of PR is symmetry; D: The funnel plot of *meta*-analysis of nephrotoxicity is symmetry; E: The funnel plot of *meta*-analysis of chest pain is symmetry; F: The funnel plot of *meta*-analysis of gastrointestinal reaction is not symmetry; G: The funnel plot of *meta*-analysis of myelosuppression is symmetry; H: The funnel plot of *meta*-analysis of fever reaction is symmetry; I: The funnel plot of *meta*-analysis of hepatotoxicityis symmetry

### 2.4 敏感性分析

为了评估分析结果的稳定性，我们进行了敏感性分析，结果显示ORR、PR、CP、肾毒性、胸痛、骨髓抑制、发热以及肝毒性比较的*meta*分析结果中逐一排除某研究重新进行*meta*分析的结果与未排除前的结果比较，改变不明显，说明其*meta*分析结果稳定可靠（图 4）。

**4:**
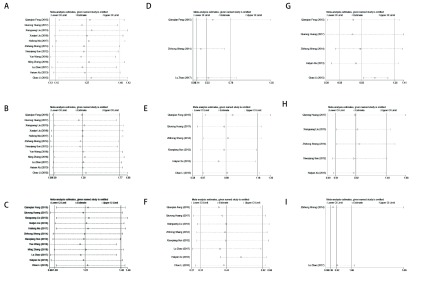
DDP与LBP胸腔灌注化疗治疗MPE疗效及不良反应的敏感性分析。A: ORR敏感性分析结果显示*meta*分析结果稳定；B：PR敏感性分析结果显示*meta*分析结果稳定；C：CR敏感性分析结果显示*meta*分析结果稳定；D：肾毒性敏感性分析结果显示*meta*分析结果稳定；E：胸痛发生率敏感性分析结果显示*meta*分析结果稳定；F：胃肠道反应发生率敏感性分析结果显示*meta*分析结果稳定；G：骨髓抑制发生率敏感性分析结果显示*meta*分析结果稳定；H：发热发生率敏感性分析结果显示*meta*分析结果稳定；I：肝毒性发生率敏感性分析结果显示*meta*分析结果稳定 Sensitivity analysis of the efficacy and adverse effects of DDP and LBP in the treatment of thoracic perfusion chemotherapy for MPE. A:Thesensitivity analysis of ORR showed that *meta*-analysis results were stable; B: Thesensitivity analysis of PR showed that *meta*-analysis results were stable; C: Thesensitivity analysis of CR showed that *meta*-analysis results were stable; D: Thesensitivity analysis of nephrotoxicity showed that *meta*-analysis results were stable; E: Thesensitivity analysis of the incidence of chest pain showed that *meta*-analysis results were stable; F: Thesensitivity analysis of the incidence of gastrointestinal reaction showed that *meta*-analysis results were stable; G: Thesensitivity analysis of the incidence of bone marrow suppression showed that *meta*-analysis results were stable; H: Thesensitivity analysis of the incidence of fever reaction showed that *meta*-analysis results were stable; I: Thesensitivity analysis of the incidence of hepatotoxicity showed that *meta*-analysis results were stable

## 3 讨论

MPE是晚期恶性肿瘤最常见的并发症之一，以胸腔积液中出现恶性肿瘤细胞为诊断依据^[22]^，胸腔积液不仅易造成肺部压迫影响患者的生活质量，还可能因胸腔积液中含有脱落癌细胞而造成肿瘤转移。若非小细胞肺癌（non-small cell lung cancer, NSCLC）患者发生MPE，根据肿瘤的亚型以及其临床分期，平均预期寿命约为3.3个月^[22]^。大量证据表明肺癌合并MPE患者的生存时间较短，而其他恶性肿瘤包括卵巢癌和不明原发病灶的恶性肿瘤等引起的MPE患者通常生存时间相对较长一些^[22]^。目前针对MPE的治疗方法主要有胸腔穿刺引流、胸导管引流、胸膜固定术和胸腔内化疗^[22]^。英国胸科协会（British Thoracic Society, BTS）指南推荐采用导管引流或胸膜固定术治疗MPE。单纯胸腔穿刺抽液的复发率高，很难彻底治疗胸腔积液，临床效果往往较差。而与胸腔内注射相比，胸膜固定术更为复杂，费用较高，少数患者给药后可能发生急性呼吸衰竭^[23, 24]^；胸膜固定术通常使用二甲胺四环素和滑石粉等，其成功率约为64%^[25]^，其胸腔积液控制率在1个月内为75%，而6个月控制率为50%^[26]^，长期效果较差；此外，胸膜固定术还伴有疼痛、发热、急性呼吸窘迫综合征等多种并发症^[27, 28]^。

胸腔灌注化疗是将化疗药物直接注射进胸腔的一种治疗方案，是治疗MPE有效的方法，可以在病灶周围形成较高的局部药物浓度从而达到治疗效果，且不会造成全身高血药浓度水平而导致严重不良反应的发生^[29]^，因而能起到明显的增效作用^[25]^。

临床实践中常采用胸腔内注射DDP治疗MPE，DDP是属于细胞周期非特异性抗癌药物，其分子量大、水溶性及渗透力强，是胸腔内局部化疗的常用药物之一，但胸膜粘连的效果较差^[30]^。1994年，肺癌研究小组（Lung Cancer Study Group, LCSG）评估并报道了胸膜内灌注DDP化疗对多种实体恶性肿瘤所致MPE患者的疗效，对标准全身治疗无效的MPE患者的总有效率仅为49%^[31]^，而DDP主要的不良反应恶心、呕吐等胃肠道反应和耳肾毒性大大地限制了其在临床上的应用。此外，DDP耐药是临床上面临的主要问题之一。

LBP是新一代铂类抗肿瘤药物，其抗癌机制与DDP相同，具有水溶性好、抗瘤活性强、与其他铂类药物无交叉耐药性及毒副作用低等特点^[8]^，且作为第三代铂类药物，LBP可以克服DDP耐药这一缺点^[32]^。王启明等^[33]^的研究显示，LBP腔内灌注化疗治疗恶性胸腔积液，主要的毒副反应为骨髓抑制及恶心呕吐等胃肠道反应。田欣等^[34]^研究结果显示LBP腔内灌注治疗MPE的有效率达80%。虽然BTS和美国国立综合癌症网络（National Comprehensive Cancer Network, NCCN）不推荐，但临床医生广泛使用DDP等抗肿瘤药物进行胸膜腔内化疗治疗MPE^[30, 31]^。在治疗肺癌方面LBP优于DDP^[32]^，然而，尚缺乏临床应用LBP治疗MPE的医学证据。

在本*meta*分析中，我们通过比较DDP与LBP胸腔灌注化疗治疗MPE的短期疗效，研究结果显示：使用LBP胸腔灌注化疗的方案在ORR、CR、PR方面均显著优于DDP方案，说明LBP治疗MPE的疗效显著优于DDP。然而由于缺乏长期生存资料的数据，二者的长期疗效尚无法进行比较。

铂类药物化疗的毒性反应也是另一被关注的焦点。Huang等^[30]^的研究显示，LBP腔内灌注化疗治疗恶性胸腹腔积液的主要不良反应为骨髓抑制及恶心呕吐等胃肠道反应，而DDP化疗对胃肠道、肾脏的毒性明显^[35]^。但尚缺乏两者胸腔内灌注化疗相关不良反应之间比较的报道。本研究比较了两者的不良反应，发现LBP胸腔内灌注化疗的胃肠道反应以及肾毒性均显著低于DDP组。本研究结果表明：LBP胸腔内灌注化疗可以明显减轻DDP化疗相关的胃肠道、肾毒性等不良反应。

本研究结果表明：与DDP比较，LBP胸腔内灌注化疗治疗MPE可以明显改善患者的ORR、CR、PR，且其肾毒性和胃肠道反应明显减轻，明显提高患者生存质量。然而，由于本研究文献数量和临床样本量均偏少，有待今后进行更大样本量的多中心随机对照的临床研究。
